# Changes in prescription drug abuse during the COVID-19 pandemic evidenced in the Catalan pharmacies

**DOI:** 10.3389/fpubh.2023.1116337

**Published:** 2023-02-14

**Authors:** Maria Perelló, Karla Rio-Aige, Pilar Rius, Guillermo Bagaría, Anna M. Jambrina, Montse Gironès, Francisco José Pérez-Cano, Manel Rabanal

**Affiliations:** ^1^Council of the Pharmacist's Association of Catalonia, Barcelona, Spain; ^2^Physiology Section, Department of Biochemistry and Physiology, Faculty of Pharmacy and Food Science, University of Barcelona, Barcelona, Spain; ^3^Institute of Research in Nutrition and Food Safety (INSA), Santa Coloma de Gramenet, Spain; ^4^Directorate-General for Healthcare Planning and Regulation, Ministry of Health, Government of Catalonia, Barcelona, Spain

**Keywords:** COVID, medicine abuse, observatory, drug, benzodiazepines, community pharmacy, pandemics

## Abstract

**Introduction:**

The impact of a pandemic on the mental health of the population is to be expected due to risk factors such as social isolation. Prescription drug abuse and misuse could be an indicator of the impact of the COVID-19 pandemic on mental health. Community pharmacists play an important role in addressing prescription drug abuse by detecting signs and behaviors that give a clearer indication that a drug abuse problem exists.

**Methods:**

A prospective observational study to observe prescription drug abuse was conducted from March 2020 to December 2021 to compare with data obtained in the previous 2 years, through the Medicine Abuse Observatory, the epidemiological surveillance system set up in Catalonia. Information was obtained through a validated questionnaire attached on a web-based system and data collection software. A total of 75 community pharmacies were enrolled in the program.

**Results:**

The number of notifications during the pandemic period (11.8/100.000 inhabitants) does not indicate a significant change compared with those from pre-pandemic period, when it was 12.5/100.000 inhabitants. However, the number of notifications during the first wave when lockdown was in place stood at 6.1/100,000 inhabitants, significantly lower than in both the pre-pandemic and the whole of the pandemic periods. Regarding the patient's profile, it was observed that the proportion of younger patients (<25 and 25–35) rose in contrast to older ones (45–65 and >65). The use of benzodiazepines and fentanyl increased.

**Conclusions:**

This study has made it possible to observe the impact of the pandemic caused by COVID-19 on the behavior of patients in terms of use of prescription drugs through analysis of the trends of abuse or misuse and by comparing them with the pre-pandemic period. Overall, the increased detection of benzodiazepines has pointed out stress and anxiety generated by the pandemic.

## 1. Introduction

Severe acute respiratory syndrome coronavirus 2 (SARS-CoV-2) was identified in Wuhan (China) in December 2019 as the cause of the illness designated as COVID-19 (1). With almost 7 million confirmed cases and more than 89,000 deaths by January 5, 2022, Spain remains one of the European countries most severely affected by the ongoing COVID-19 pandemic (2–4).

In Spain, the first virus case was detected on January 31, 2020, and several weeks later, on February 25, 2020, the first case in Catalonia (a northeastern region in the country) was identified after a 36-year-old woman visited Italy from February 12 to 22, 2020 (5).

From then on, the adoption of lockdown measures by the national and regional governments steadily increased over time, from the recommendation of preventive measures in late February and early March to increasingly stricter social distancing measures. On March 13, a nationwide lockdown was announced and on March 15 it was enforced. In addition, a strengthened lockdown was rolled out with the closure of all non-essential economic activities on March 31 (6).

On April 25, Spain started to ease its lockdown with a gradual lifting of restrictions due to decreasing trends in confirmed cases, hospitalizations, and daily deaths. Hence, the “lifting lockdown” process began and the state of emergency ended on June 21. Although the situation had stabilized by the summer period (July-September) and a significant rise in the number of COVID-19 cases was not observed, the Government of Catalonia forbade gatherings of more than 10 people in public or private premises and advised people to stay at home unless strictly necessary.

In October, the second wave started and the state of emergency was rolled out again. This new one ran from October 25, 2020, to May 9, 2021. As in the first state of emergency, the level of restrictions varied depending on the successive waves of COVID-19 cases: The second wave was from October to early December; the third wave from January to March, and the mild fourth wave during April and May. Soon after the first COVID-19 vaccine (BNT162b2 mRNA) was approved in December 2020, Spain started its mass immunization campaign.

In this context, an impact of the pandemic on the mental health of the population is to be expected due to risk factors such as social isolation, uncertainty over disease status, and economic and housing problems (7–9). The pandemic also affects the control of chronic disease (10). Additionally, a number of studies have shown that fear of COVID-19 infection was associated with high levels of emotional stress, especially in women, while an increase in anxiolytics use during lockdown was observed (7). Furthermore, it is reported that being young had a positive association with depression and anxiety (7, 8, 11). Although the impact of the COVID-19 pandemic on mental health cannot be quantified yet, there is the suggestion of a wave of mental illness associated with the consequences of the pandemic (12) and a rise in the consumption of benzodiazepines to cope with these disorders (7, 11, 13–16).

Concerns were also reported by several sources and some experts described an increased availability after the lockdown period of diverted prescription opioids, such as tramadol, buprenorphine and methadone (17).

As a vital part of the healthcare system, pharmacies play an important role in providing medicines, therapeutics, vaccines, and critical health services to the public. Moreover, pharmacists have knowledge about the safe and effective use of medications and about the adverse effects of their inappropriate use. In addition, pharmacists do more point-of-care work to help take the pressure off doctors. Patients have also turned more often to the pharmacist to request chronic medication and/or that necessary to tackle the situation generated by the pandemic (18–20).

Equally, prescription drug abuse and misuse, defined as the intentional use of a medication without a prescription or in a way other than as prescribed, or for the experience or feeling its causes, could be a possible indicator of the COVID-19 pandemic's impact on the behavior of the public due to effects on mental health. Furthermore, the inappropriate use, can be also unintentional, such as when it is due to ignorance or cognitive impairment (21).

According to the National Survey on Drug Use and Health (NSDUH), in 2021, an estimated 9.6% of past year users of drugs other than alcohol (or 10.2 million people) perceived that they used these drugs “a little more or much more” during the COVID-19 pandemic than they did before, which include prescription pain relievers, tranquilizers, stimulants, or sedatives (22). As well, in Europe, it is estimated that at least 5 800 overdose deaths, involving illicit drugs, occurred in the European Union in 2020, this represents an estimated mortality rate due to overdoses of 17.4 deaths per million for the adult population. Most of these deaths are associated with polydrug toxicity, which typically involves combinations of illicit opioids, other illicit drugs, medicines and alcohol. In some countries, benzodiazepines are commonly mentioned (23).

Given this situation, the aim of the work was to identify trends of medicine abuse in Catalonia and study whether there has been any change in the pattern with respect to previous years, through report of community pharmacies.

For this purpose, the information was obtained from the Medicine Abuse Observatory (MAO), that has been operating in community pharmacies of Catalonia since 2017, and allows to observe and analyze the behavioral patterns of the population with respect to this phenomena (24).

## 2. Material and methods

### 2.1. Community pharmacy framework

As community pharmacists have a key role in carrying out epidemiological surveillance and should be committed to promoting the safe and effective use of medicines, in 2017 the Medicine Abuse Observatory (MAO) was set up in Catalonia. The MAO was settled as a project supported by the Catalonia Pharmacists Council and the Ministry of Health of the Government of Catalonia. It makes it possible to observe and analyze trends about the most diverted drugs and the behavioral patterns of the population with respect to this issue *via* community pharmacies. In this context, we conducted a prospective observational study from March 2020 to December 2021, taken as the COVID-19 period, in order to compare it with data from 2 years prior (July 2017-February 2020), taken as pre-COVID-19 period (22).

### 2.2. Enrolled pharmacies

Data was obtained from community pharmacies enrolled in surveillance for the detection of suspected cases of drug abuse and misuse project. There were 60 of them during the pre-COVID period and this number rose up to 75 for the COVID period. In both periods, selected pharmacies belonged to the Catalan sentinel pharmacy network (Catalan Sephanet) (25). The pharmacies are scattered throughout the region, based in 3 phases. First, a selection phase, to determine the minimum number of sentinel pharmacies and the location of these to obtain the greatest possible representativeness. This phase includes a cluster and population analysis, and adjustment for strata and population. Second, a voluntariness and random selection phase of the pharmacies that belong to the selected area, to mitigate bias in the reporting depending on the degree of motivation. Third, a training by the Barcelona College of Pharmacists, in order to standardize data collection. The main topics covered were the basis of the method and operational procedures coupled with a theoretical framework furnishing information about the phenomenon. The training sessions were performed regularly to resolve issues and clarify questions about screening procedures (24, 25).

### 2.3. Data report

A validated questionnaire (Abuse Drug Questionnaire, ADQ) was created. It was based on Finch's criteria, that enables to identify signs and behaviors that give a clearer indication that a drug abuse exists. These elements, which Finch described in 1993, are: pattern of calling for refills after hours and/or repeatedly needing early refills, prescriptions from multiple physicians, frequent visits to emergency rooms, strong preference and knowledge for a particular drug and incongruence between severity of the complaint and the physical presentation. Based on this theoretical framework, the situations that would arise in community pharmacies, such as repeated requests for medicine, or the request for a prescription medicine with a false prescription or without it, would be indicators of suspected misuse or abuse of these medicines.

The questionnaire consisted of an anonymous multiple-choice test containing 10 closed and two open-ended questions categorized in four different parts. Pharmacist identification (questions 1 and 2). Patient demographic variables as age, sex, and origin, are included in questions 3 to 5. The substance involved and how it is requested, was required in questions 6 to 9. In this sense, it is considered “does not require a prescription” for over the counter (OTC) medicines and “requested with prescription” for prescriptions needing frequent refills and/or from multiple physicians. We consider “probably forged prescription” as a counterfeit prescription (copies) or any falsification made on a right prescription form. Finally, in question 10 and the 2 open-ended questions, pharmacist management is enquired. The aim of these three questions is to know in which cases the medicines are dispensed and to study the reasons for which these medicines are dispensed.

The pharmacist filled out the questionnaire when a patient that requested a medicine presented two or more of the defined signs and behavioral symptoms and was suspected of being a medicine abuser. Patient information was obtained anonymously by observation during the interview and neither verbal nor written consent were needed. Otherwise, the substances to follow up were chosen taking into account the evidence from scientific literature and data from our environment (22). In this sense, a list to select the type of benzodiazepines was included and also an item entitled “others” that allows pharmacists to report any medicine.

In order to ease the reporting, the ADQ collected during the studied period were passed by a web-based survey. Data collection software called Typeform (Typeform SL, Barcelona, Spain) was embedded in the Barcelona College of Pharmacists' website, which is the principal online work tool for pharmacists in this area of Spain. This software transformed the ADQ electronic data into an Excel spreadsheet to operate them.

### 2.4. Statistical analysis

To categorize the number of notifications of both periods, the number of inhabitants corresponding to the enrolled pharmacies for each one was considered: 146,335 for the data coming from the 60 community pharmacies in the pre-COVID period and 218,701 for the data coming from the 75 community pharmacies in the COVID period. Likewise, the categorical variables obtained for the COVID period were analyzed as percentages and compared with those obtained in the pre-pandemic period. The X^2^ test was used for this purpose and a *p*-value < 0.05 was considered statistically significant. Additionally, quantitative analysis was also performed for some items such as benzodiazepines. The analyses were conducted with SPSS software, version 18 (SPSS Inc., Chicago, IL, USA).

Multiple correspondence analysis (MCA) was also performed to find similarities in the individual profiles simultaneously by R version 4.1.2 (R Foundation, Austria) (https://www.R-project.org/) using the packages FactoMineR (https://cran.r-project.org/web/packages/FactoMineR/index.html) for the analysis and factoextra for the visualization (https://cran.r-project.org/web/packages/factoextra/index.html). In this analysis, two categories that present high coordinates and are close in space are directly associated with each other. When the cos2 value for one variable category is close to one, this indicates it is well represented by two dimensions. This MCA analysis made it possible to find similarities between customers in terms of their characteristics and behavior and establish the relation and the degree of association between different variables.

## 3. Results

### 3.1. Number of notifications

The number of notifications during the pandemic period was 11.8/100,000 inhabitants in the Catalonian region and this proportion was not significantly different when compared to the pre-pandemic period (12.5/100,000 inhabitants, *p* = 0.39). It should be borne in mind that the pandemic period consisted of a number of periods in which only the first wave included total lockdown. Thus, the number of notifications during the first wave was 6.1/100,000 inhabitants, significantly lower than in both the pre-pandemic period and the whole pandemic periods (*p* < 0.05).

**Figure 1:**
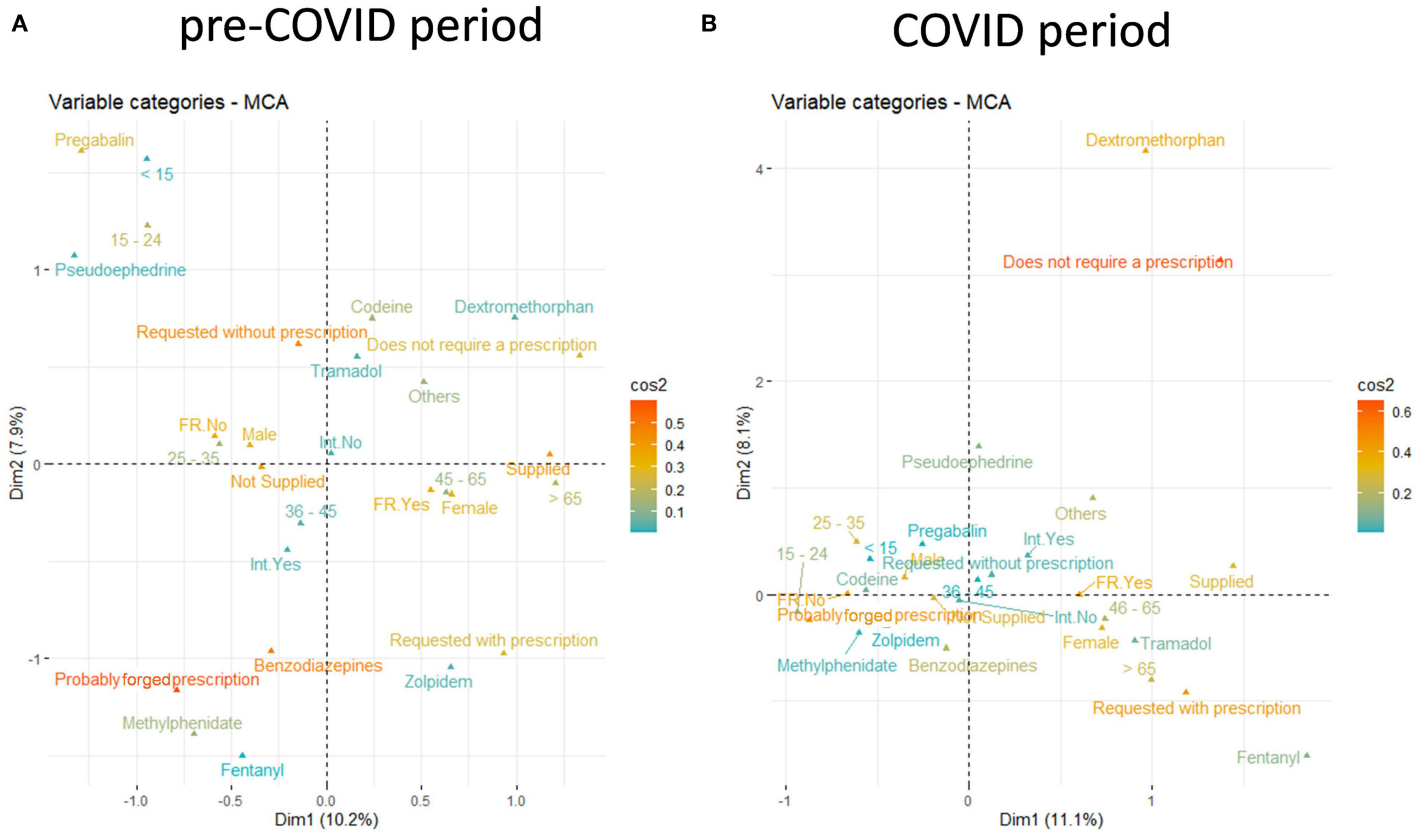
Two-dimensional MCA plots for the pre-COVID period **(A)** and the COVID period (**B)**. Cos2 measures the degree of association between variable categories and axial categories. If the variable category is well represented by the dimensions, values for cos2 are close to one.

All the validated notifications under study allow to characterize the patient's profile, the substances involved, the drug requested, and the supply of medicine. First, the participant's features were multi-parametrically approached to analyze similar profiles between the individuals in the study and to evaluate associations between variable categories (Figure 1) by multiple correspondence analysis (MCA). The variance obtained was 10.2% for Dim-1 and 7.9% for Dim-2 in the pre-COVID period, while the explained variance for the COVID period was 11.1% for Dim-1 and 8.1% for Dim-2 (Supplementary Figures 1A, B, respectively). The variables had similar correlations with the first two dimensions in both periods, where the REQUEST variable was the most correlated with Dim-1 and the second most correlated with Dim-2, followed by the DRUG variable, the most correlated with Dim-2 (Supplementary Figures 1C, D). This finding was in line with the visualization of the results in Figure 1 in which it can be observed that some REQUEST categories had the highest cos2 value, “Probably forged prescription” in the pre-COVID period (Figure 1A) and “Does not require a prescription” (Figure 1B) in the COVID period, indicating that these factors were important in explaining the variability in the dataset. Moreover, it can also be seen that “Probably forged prescription” and “Benzodiazepines” were very close and appear on the negative y-axis (Dim-2) in the pre-COVID MCA (Figure 1A), indicating a strong correlation between them. On the other hand, these categories were less representative in the COVID MCA, where the categories “Does not require a prescription” and “Dextromethorphan” gained strength on the positive y-axis (Dim-2) (Figure 1B). In line with these results, when individuals were colored by variables, the REQUEST variable tended to cluster the subjects (Supplementary Figure 2). These overall differences between periods can be better observed when they are quantitatively stratified by factors.

### 3.2. Change in the patient profile during the pandemic period

**Figure 2:**
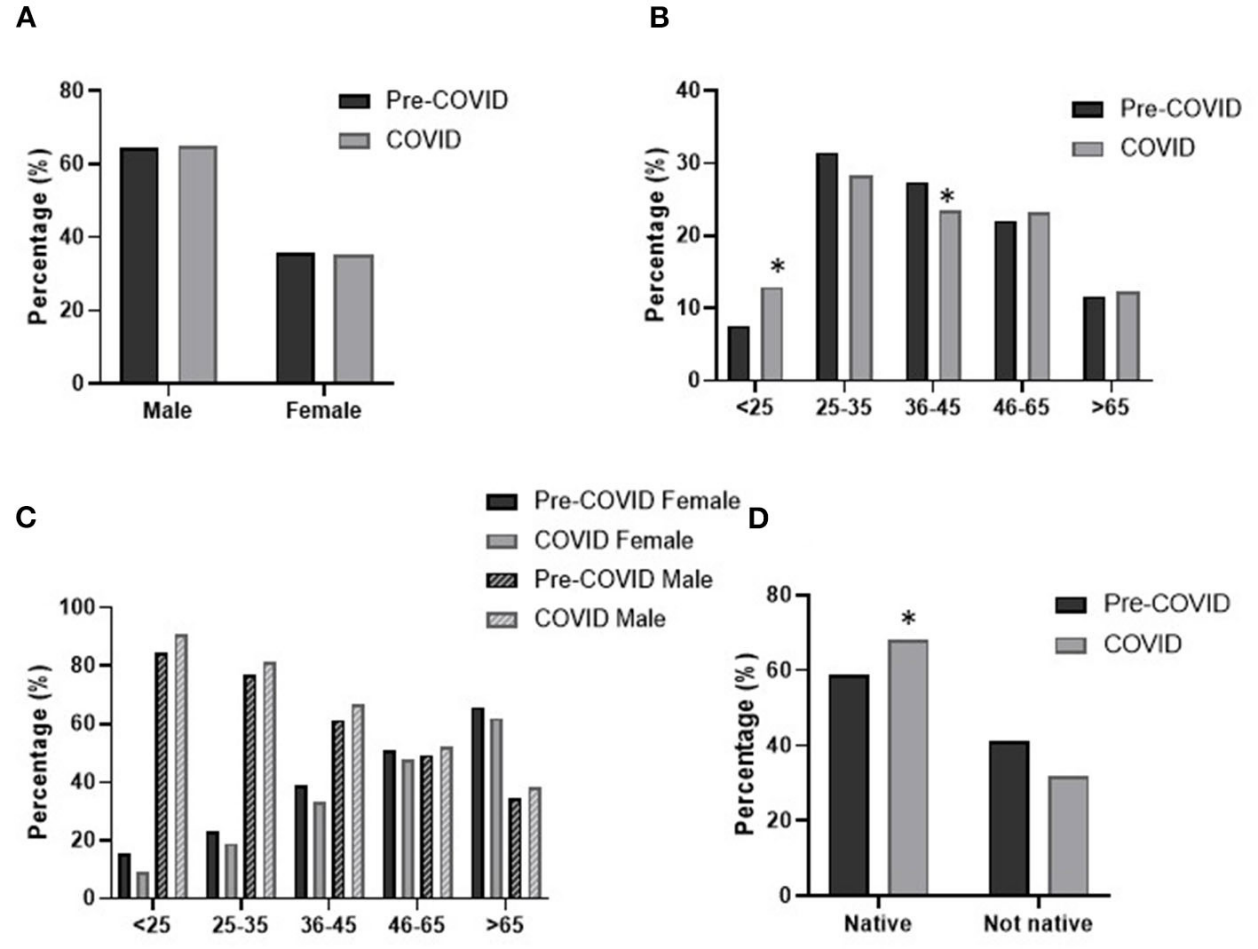
Patient profile across pre-pandemic and pandemic periods. Distribution of participants by **(A)** sex, **(B)** age, **(C)** age and sex and **(D)** origin. Statistical differences: *p < 0.05 pandemic vs. pre-pandemic.

The proportion of male and female involved in the notifications was similar between the two periods studied, with male in the majority (~65% male: ~35% female) (Figure 2A).

In relation to age, in both periods the highest proportion of notifications was from patients in the 25–35 years range (~30%) followed by those aged 36–45 and 46–65, both with very similar values (22–27%) (Figure 2B). The lowest proportion of prescription drug users was found in the youngest and the oldest age intervals (<13% in both cases and periods). When both periods were compared, we observed that during the pandemic there was a significant rise in the proportion of users in the youngest group (<25 years) at the expense of those in the 36–45 years range (*p* < 0.05).

The combination of age and sex data makes it possible to better characterize the profile of the patients showing a differential predominance pattern between male and female. In the most prevalent age range (25–35 years) as in the <25 years and 36–45 years ranges, there are more male than female (~80% vs. 20%). In addition, this pattern changes and the proportion of female increases in the oldest group (>65 years). The 46–64 years age group has similar proportions of male and female (~50%). However, no significant differences were observed between the pandemic and pre-pandemic periods (Figure 2C).

Also, in relation to whether the notifications concerned a native or non-native patient, and although in both cases there were more native patients involved (~60–70%), in the pandemic period there was a rise in native patients asking for an item considered drug abuse in pharmacies (*p* < 0.05) (Figure 2D).

### 3.3. Pandemic effect on the involved substances and drug requests

**Figure 3:**
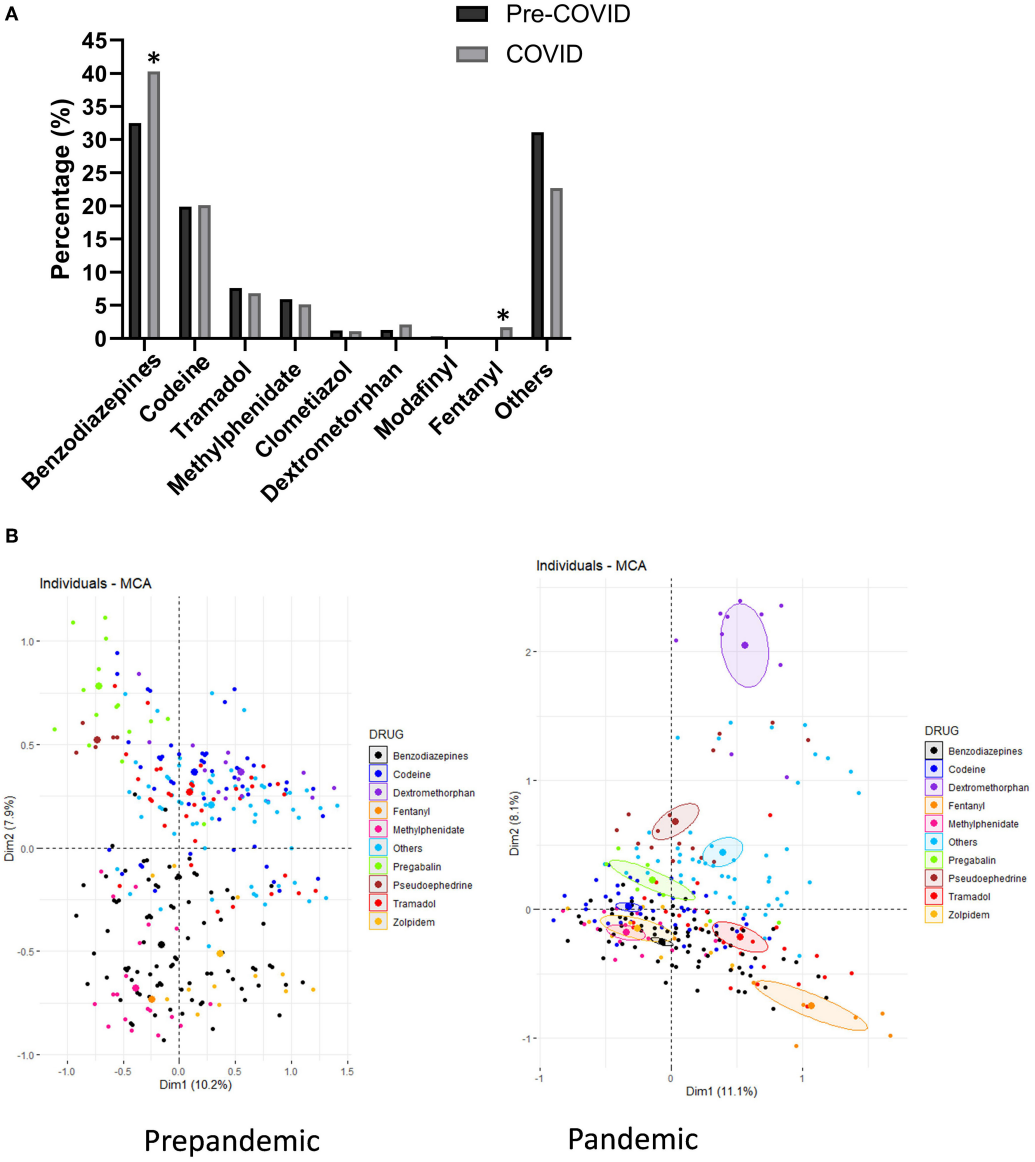
Description and proportion of type of drug according to **(A)** both periods and **(B)** depiction by MCA analysis. Statistical differences: *p < 0.05 pandemic vs. pre-pandemic.

The data obtained in both the pre-pandemic and pandemic periods show that the substances involved are quite variable albeit with a similar preliminary pattern (Figure 3A). The main drug class involved was benzodiazepines (~30–40%), followed by codeine (~20%), tramadol (~7%), and methylphenidate (~5%), while the rest of the requested prescriptions are present in the questionnaire at <2%. However, other not explicitly requested drugs were also involved in an overall proportion of ~25%. Even so, there was a significant increase during the pandemic in the detection of benzodiazepines and fentanyl (*p* < 0.05). In addition, a different pattern was found in some drugs stated in the “others” section. For instance, pseudoephedrine was requested during the pandemic by 4.9%, pregabalin by 1.8%, and cycloplegic drops by 0.2% whereas in the pre-pandemic period these values were about 0.8, 5.8, and 4.4%, respectively (*p* < 0.05). This differential shift in patients' behavior on drug substances is clearly illustrated by the MCA in Figure 3B.

Regarding benzodiazepines, the group of prescription drugs most frequently detected, it was observed that the number of notifications intended to increase during the pandemic and it rose from 3.95/100,000 inhabitants to 4.93/100,000 inhabitants (*p* = 0.07). The most detected benzodiazepine was clonazepam (44.2%) followed by alprazolam (22.5%), lorazepam (17.4%) and diazepam (10.5%).

The sex variable also shows no difference between periods and they were higher in males in both periods.

In terms of age, benzodiazepines represent the most detected group of medicines in >65 years (56.3% of notifications) with no difference between periods. Despite this, the <25 group had a significant increase in incidence (*p* < 0.05) and, in contrast, the 25–35-year-old group showed a decrease (*p* < 0.05). Benzodiazepine use by the rest of the age ranges did not vary due to the pandemic (data not shown).

**Figure 4:**
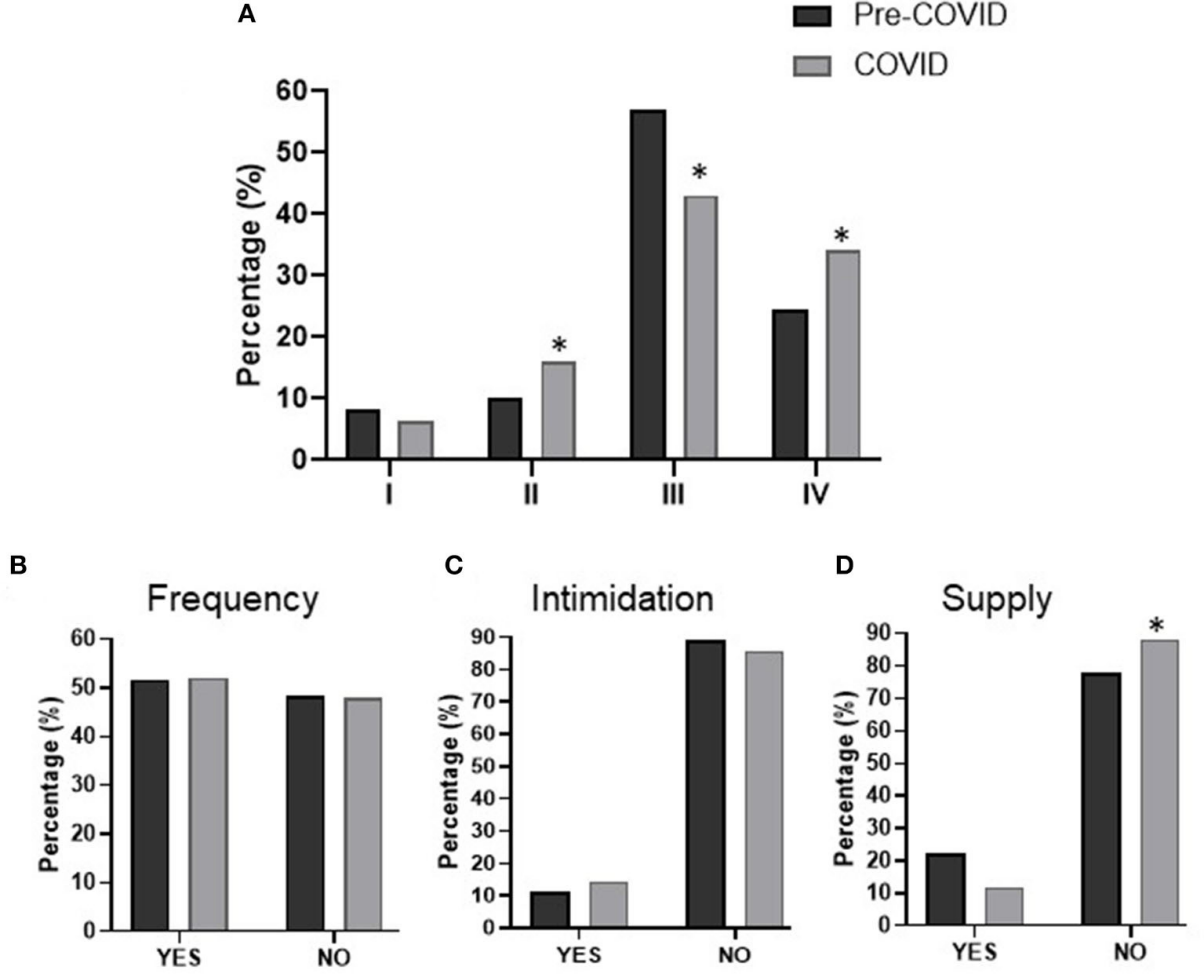
Drug-requesting behavior of patients. Distribution of participants according to **(A)** type of request, **(B)** frequent request, **(C)** use of intimidation, and **(D)** supply of medicine. Requesting types are as follows: I. prescriptions corresponding to an over-the-counter (OTC) drug; II. formal prescription; III. without prescription; IV. Forged prescriptions. Statistical differences: *p < 0.05 pandemic *vs*. pre-pandemic.

With reference to the drug request or the way users tried to get the medicine, some changes due to COVID were observed (Figure 4A). In both the pre-pandemic and the pandemic periods, most of the notifications were due to drugs requested without a prescription (~50%) and forged prescriptions (~30%), whereas notifications coming from over the counter (OTC) drugs and formal prescription drugs were less frequent. The pandemic triggered a significant rise in requests with a prescription and also with forged prescriptions (*p* < 0.05). In addition, in the COVID period the number of notifications of medicines that required a prescription or which were requested without one was also lower (p<0.05). Notifications of OTC drugs behaved similarly in both periods.

The benzodiazepine request pattern remains practically the same in both periods, where ~50% of people try to get the medicine by using a forged prescription, ~27% by requesting without a prescription, and ~20% through a formal prescription (data not shown).

### 3.4. Changes in request frequency and supply of medicines

The request for the medicine was sometimes performed insistently, and this frequency pattern was similar in both periods (Figure 4B). In certain cases, the drug was asked for using intimidation. It should be noted that a trend to increase (from ~11 to ~14%) was observed during the pandemic period vs. the pre-pandemic one, but it was not statistically significant (*p* = 0.10) (Figure 4C).

The management and attitude of pharmacists when addressing these requests was similar in both periods and in ~80% of cases dispensation did not occur. However, the supply percentage was slightly lower during the pandemic period (*p* < 0.05) (Figure 4D).

Behavior related to benzodiazepines showed no difference between periods and request frequency was around 50% in both. In addition, no difference related to intimidation was detected either. It could be observed that nearly half of notifications with intimidation involved benzodiazepines (50.7% for the pre-pandemic and 46.9% for the pandemic period).

As to the management of pharmacists when abuse or misuse was under suspicion, during the pandemic period fewer supplies were delivered (*p* < 0.05) and 60.6% of them were so to people aged 46–65 and >65 years, who made requests with higher frequency (*p* < 0.05 for the 46–65 and >65 years groups vs. the other age-groups), and mainly were anxiolytics and opioid painkillers (fentanyl, codeine, and tramadol). However, there was overall a decrease in the dispensation of benzodiazepines (*p* < 0.05) to people in this age range.

### 3.5. Waves of the pandemic period

**Figure 5:**
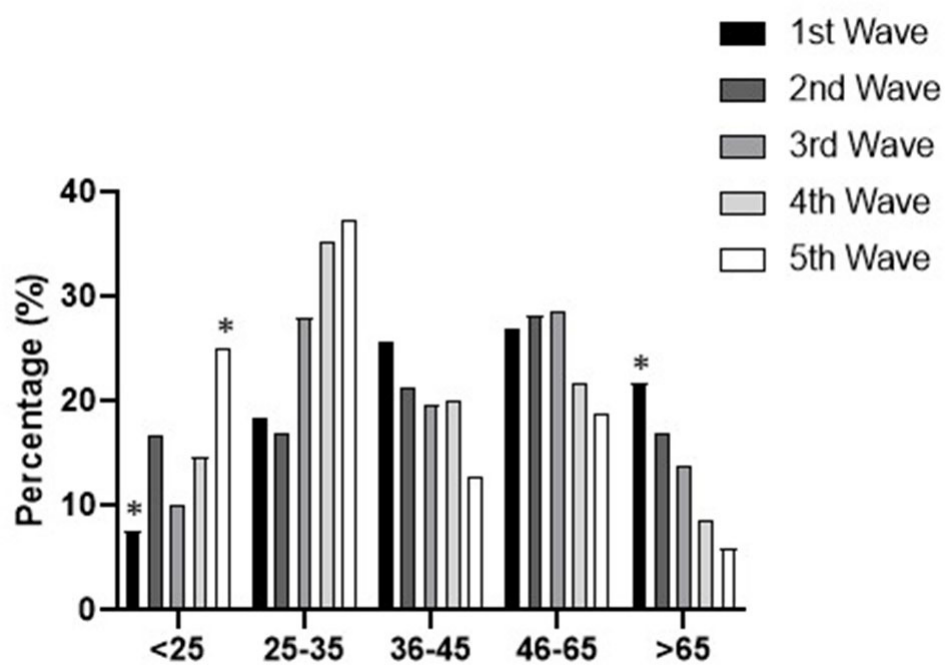
Patient age distribution profile according across waves. Statistical differences: *p < 0.05 vs. the value in the total pandemic.

Although the global period of the pandemic has been analyzed as a whole, we are aware that each wave within this period had a different restriction pattern in which the first wave was the most rigorous as there was total lockdown of the population. Thus, after analyzing the patterns in each wave, we found similar behavior in some aspects, but some trends and statistical differences appeared too, especially with respect to age distribution (Figure 5).

On the one hand, as noted above, the number of notifications per inhabitant was similar across waves, with the exception of the first wave, the only one including total lockdown and showing significantly lower numbers (6.1/100,000 inhabitants) than the rest of the pandemic periods, which were even lower than the pre-pandemic period (*p* < 0.05). Besides, the patient profile did not change regarding sex, and the male-female interval was similar across waves (from 55–45% to 74–26%). However, when the influence of age in the notifications was studied, a pattern over the course of the pandemic waves (*p* < 0.05) was found, whereby younger patients (<25 and 25–35) rose for some variables at the cost of older ones (45–65 and >65) (Figure 5).

No changes were found between waves from the native/non-native patient point of view, where the maximum differential proportion was 82.4%/17.6% in the first wave, shifting to 62.2%/37.8% in the last wave (*p* < 0.05). As for drug requests and patient intimidating behavior, a similar pattern was also observed over the course of the pandemic. However, the request frequency proportion was different across the waves of the pandemic, inasmuch as request notifications were more frequent in the first three waves (64.9, 56.4, and 62.6%) than in the last two (44.4 and 38.9%) (*p* < 0.05). Nonetheless, pharmacist supply did not change during the COVID period analyzed. As for the type of drug, the contribution of each one was variable among waves, with no clear pattern during the whole pandemic period, and in all cases, there were higher contributions of benzodiazepine and fentanyl prescription than during the pre-pandemic period (*p* < 0.05).

## 4. Discussion

This study describes changes in prescription drug abuse trends during the COVID pandemic. Results are in line with preliminary findings which, despite not supporting a rise in drug use during the early stages of the COVID pandemic (16, 26), do show an increase in some prescription drugs such as benzodiazepines (14–17). This may be accounted for by users wishing to combat the concerns and anxiety experienced in response to the COVID-19 pandemic and the resulting lockdown measures. The rise in prescription medicines used to cope with increased mental health issues during the COVID-19 pandemic is also highlighted by the European Monitoring Center for Drugs and Drug Addiction (EMCDDA), which refers to loneliness, feeling depressed and stress as the main reasons for increased use of benzodiazepines (27).

In addition, the COVID-19 pandemic has affected drug markets (supply shortages of drugs as well as price increases). These problems, combined with a general economic loss, can lead to changes to prescription or OTC medicines that are mixed with cheaper drugs (14, 26–28).

Equally, primary healthcare assistance reduced mental health services during the national lockdown due to the COVID-19 outbreak and there was a decrease in follow-up and control of chronic diseases and adult vaccination coverage (10). The possible consequences of these deprivations include an increase in admissions to emergency departments for mental illness and drug and alcohol use and also a rise in suicide risk (15). In fact, according to the European Drug Report, an increase in emergency room visits related to benzodiazepines was detected in a sample of surveillance hospitals in 2020 compared to 2019 (29).

Although the consequences in terms of increases in dependence on substances such as alcohol or prescription drugs may only become visible with time, our study makes it possible to observe the behaviors and medicines that have been most identified in relation to this phenomenon in community pharmacies.

First of all, in terms of age, and even though the highest proportion of users were patients in the 25–35 years range, there was a rise in those under 25 years of age and also in those over 65. This increase in the extreme ages can be explained, firstly, by the change in the behavior of drug markets as noted above, and secondly, by the lack of monitoring of the health status of the older adults by primary care services. This is consistent with data in a study carried out in the United States, where it was reported that early adolescent substance use during the pandemic was associated with increased use of nicotine and misuse of prescription drugs (30), and also with concerns about possible increases in drug-related deaths in some countries, mostly among young people and due to intake of benzodiazepines (16).

Turning to sex, in this analysis males asked for abuse drugs more often than females, which is in line with the published evidence on this phenomenon. The combination of age and sex data in our study shows that males are more frequent in the 25–35 years age group, while women are more common in the oldest group. Also, if we consider the medicines involved and the sex of patients, there's an association pattern for the COVID period between tramadol and fentanyl with females. This contrasts with the data obtained in the previous study during the pre-pandemic period, where no such association was observed, probably because women and young people had more mental health issues during lockdown (7).

There was an increase in the use of benzodiazepines, the most common substance, in all age groups, but it is especially remarkable in the group over 65 years of age. Our results are in line with the massive increase in reports of abuse during the pandemic, which seem to point at social isolation as one of the greatest risk factors for their drug abuse (31). Indeed, a recent study about the use of drugs and health resources by patients with pre-existing mental disorders (depression or anxiety) pointed out that there was a remarkable increase in the number of daily doses per inhabitant (DDI) of some benzodiazepines such as alprazolam or lormetazepam during the six months following the lockdown end in a regional health service in Spain (13). These data tie in with those published by the Spanish Agency of Medicines and Medical Devices, which reported that in the first quarter of 2020, the DDI of these substances was higher than in the last quarter of 2019 (57.19 DDI *vs*. 55.51 DDI) (13).

In this approach, tramadol was most commonly detected as an abuse drug substance in the 46–65 age group, and Z-drugs in older age groups. In contrast, the substance that was widely detected in the 25–35 years of age group was pseudoephedrine, which has sympathomimetic properties and whose acute effects include stimulant effects such as euphoria, lower sense of fatigue, anorexia, accelerated thinking, and psychotic symptoms with auditory and visual hallucinations. Oral and intravenous use has been recorded in misuse cases (32). It is also used as a slimming agent and in sports as an ergogenic agent. Moreover, there is a growing interest in preparations containing pseudoephedrine related to their use for recreational purposes, especially by adolescents and young adults, and for the production of psychoactive substances—the synthesis of methamphetamine and methcathinone (ephedrone) used as designer drugs (33). This high demand needs to be closely monitored and scrutinized in terms of its health and social consequences. Here Project STOP, a decision-making tool for pharmacists developed in Australia, allows community pharmacists to verify pseudoephedrine requests, and since its implementation there has been a downward trend in pseudoephedrine sales. This program is a multi-strategic approach to reducing inappropriate supplies through community pharmacies (34). This study also notes that community pharmacists are healthcare professionals who can prevent the potential misuse and abuse of medicines and also provide the option to monitor trends in this phenomenon.

Experts working in harm-reduction services in some EU Member States have also suggested that the use of methamphetamine may have become more popular in some user groups (35).

In the case of codeine, the same trend has remained and has been more commonly reported for younger (<25 years) people. However, in this age group, there has also been an increase in the notifications of benzodiazepines, which is related to the higher involvement in the detection of this phenomenon in young people by community pharmacies. The rising trend of misuse of benzodiazepines as well as of other substances that indicate illicit use needs to be monitored (16).

Apart from these substance-related data, the positive impact of the lockdown imposed due to the state of health emergency in order to curb the disease reduced people's mobility, including their access to pharmacies. Therefore, the large difference between the number of requests made by native patients vs. non-native patients can be associated with this situation and the restrictions enforced which reduced the percentage of non-native patients involved (36). As a result, the number of notifications per 100,000 inhabitants is lower during the pandemic lockdown period compared to the pre-pandemic period.

Thus, the pandemic meant that people could not seek medical attention whenever needed. This prompted the Health Ministry to allow pharmacies to supply the medicine even when the prescription had expired (10). At the same time, patients tried to abuse some medicines because they did not have enough, meaning that they were consuming more than in the pre-pandemic period. This may reinforce the data obtained related to the request type, in which there was an increase for some medicines with a prescription. This situation was especially common in the older age groups, who were also more associated with requests involving intimidation. In fact, in the early stages of the pandemic, verbal intimidation in requesting drugs rose up to significantly different values compared to pre-pandemic levels. Although as the pandemic went on this behavior tended to revert to its usual pattern, there was nevertheless a clear trend in threatening behaviors, which might have been related to stress and fear due to the circumstances. By contrast, young patients did not confront pharmacists but rather tried to get the drug through forged, copied or altered prescriptions or by going to different pharmacies.

Here it should be noted that pharmacists deliver services that have been shown to improve patient outcomes by providing information on COVID-19 and dispensing medications to maintain continuity of healthcare along with other services, so they remain the most accessible healthcare provider. However, pharmacists also experienced an increase in the number of patients seen during the pandemic. It is up to pharmacists to reassure patients and provide care while taking into account their mental health. In addition, the increase in the burden of pharmacist duties may also have led to a decrease in the pharmacist's mental wellbeing, as some studies suggest (37, 38). However, and in spite of this, pharmacists have continued to provide good healthcare to patients and detect patterns of probable abuse or misuse of medicines. Consequently, they have refused to supply these medicines when not indicated, and the number of dispensations made even decreased compared to the pre-pandemic period.

Community pharmacists play a vital role in supporting local communities, providing reliable information to patients, and relieving pressure on the rest of the healthcare system. Nonetheless, progress needs to be made in other demands not related to SARS-CoV-2 infection and it is essential to reestablish all care activity that has been interrupted as soon as possible. Identifying mental health-related needs is a priority along with regular assessment of substance use and suicidal ideation to evaluate the prevalence of psychological distress over time (10, 14, 39). This is in line with what Nora Volkow, the Director of the National Institute on Drug Abuse (NIDA), has said: “Clinicians should monitor for signs of substance misuse or use disorders in their patients, given the unprecedented stresses, fears, or even grief they may be facing” (40).

Regarding the various waves that took place during the pandemic, the greater detection and involvement of older people in the first period and the higher prevalence in frequently requesting some medicines probably reflects their concerns about the situation. They may have feared running out of medication, bearing in mind that specifically in the first wave the population was completely locked down. So they sought to have sufficient treatment to cope with the distress and nervousness produced by the pandemic. Hence as the pandemic progressed and restrictions and accesses were mitigated, a decrease in the involvement of the older adults was observed contrasting with higher detection of young people seeking to get medicines, most likely for the reasons already discussed above. Thus, the last period showed an increase in requests for medicines that do not need a formal prescription, specifically dextrometorphan and some sympathomimetic agents used as decongestants. This trend is related to this younger age group who also tried to get other drugs using forged prescriptions or by asking for them with no prescription.

## 5. Conclusions

This study made it possible to observe the impact of the pandemic caused by COVID-19 as shown on the behavior of patients regarding the use of prescription drugs through analysis of the trends of drug abuse or misuse and comparing them with the pre- pandemic period.

Overall, the first wave was the period with the highest impact, and a rise in benzodiazepines points to stress and anxiety generated by the pandemic and evidencing use of substances to cope with those feelings. A higher frequency of younger and older age groups showed that the needs of these population segments were greater, since published data suggests the pandemic aftermath has affected them the most. Efforts and resources need to be provided where they are due so that they are beneficiaries of the best healthcare.

## Data availability statement

The raw data supporting the conclusions of this article will be made available by the authors, without undue reservation.

## Ethics statement

Ethical review and approval was not required for the study on human participants in accordance with the local legislation and institutional requirements. Written informed consent for participation was not required for this study in accordance with the national legislation and the institutional requirements.

## Author contributions

Conceptualization: MP, MR, and FP-C. Methodology: MP, MR, FP-C, and MG. Software: KR-A. Validation: GB, PR, and AJ. Formal analysis: MP, KR-A, and FP-C. Investigation: MP and MR. Resources: MP, PR, and MR. Data curation: MP and KR-A. Writing—original draft preparation: MP, FP-C, MR, and KR-A. Writing—review and editing: MP, PR, and AJ. Visualization: MG and PR. Supervision: MR and FP-C. Project administration: MR, PR, and GB. Funding acquisition: MR. All authors have read and agreed to the published version of the manuscript.
