# Supplementary material for: Changes in prescription drug abuse during the COVID-19 pandemic evidenced in the Catalan pharmacies
Source: Front Public Health. 2023 Feb 14;11:1116337. doi: 10.3389/fpubh.2023.1116337 (PMC9971931; doi:10.3389/fpubh.2023.1116337)
Supplement: Supplementary file 1 [file Data_Sheet_1.PDF]

Supplementary Figure 1

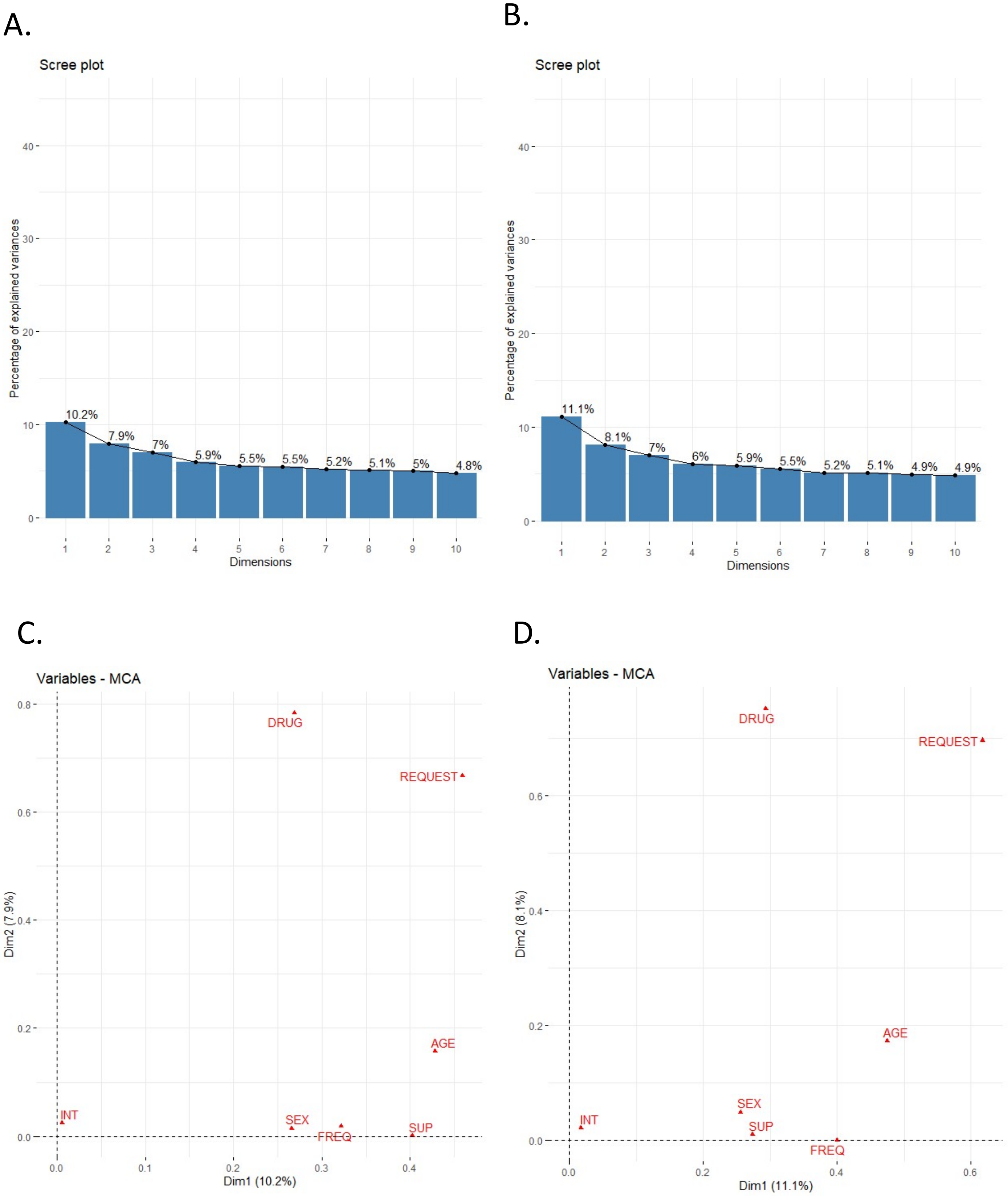

Supplementary Figure 1. The proportion of variances explained by the different dimensions in the pre-COVID period (**A**) and in the COVID period (**B**). Correlations between variables and principal dimensions in the pre-COVID period (**C**) and in the COVID period (**D**).

Supplementary Figure 2

A. **Pre-COVID period** **COVID period**

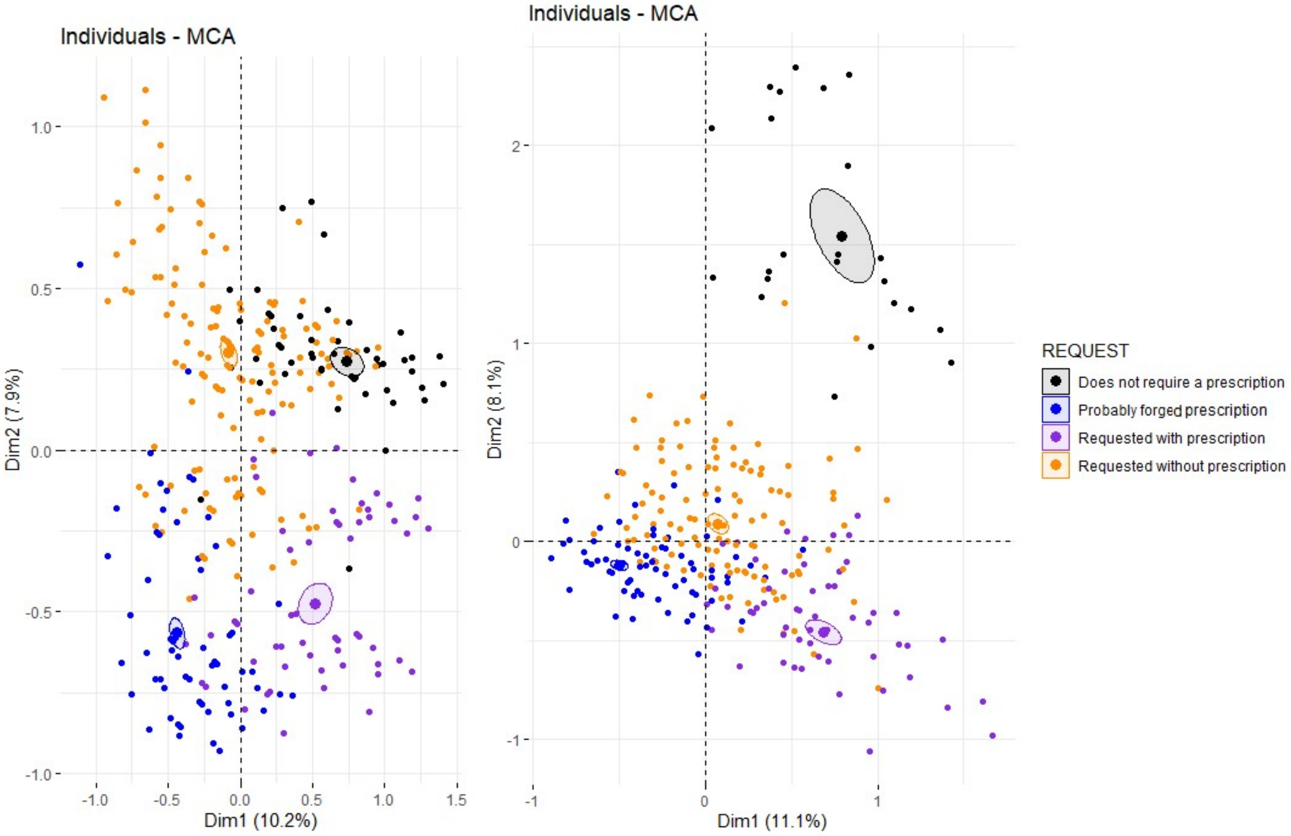

Supplementary Figure 2. Two-dimensional MCA plot with the subjects coloured regarding the levels of the variable "REQUEST" in the pre-COVID period (**A**) and in the COVID period (**B**).
